# Pathology of Neuralgia

**Published:** 1860-06

**Authors:** L. D. Robinson

**Affiliations:** New Elizabeth, Ind.


### PATHOLOGY OF NEURALGIA.

By L. D. ROBINSON, M. D., of New Elizabeth, Ind.

Neuralgia is one of the most painful as well as interesting affections with which the physician has to deal; and the true pathology of the disorder seems very illy understood by many of our standard medical authors. Indeed, I believe none of them profess to plainly and positively elucidate its pathology. We think the profession has a *rationale*—a true pathology—and one that can be plainly elucidated, and substantiated by good and sufficient authority.

Eberle says, "in many instances neuralgic affections are nothing more than masked agues from the influence of koino-miasmata." Again, he says: "It may, I think, be assumed as a fact, that neuralgia may depend upon different causes—on local inflammation or congestion of the affected nerve—on organic disease of the brain—and most commonly on a sympathetic irritation, from latent irritation in other parts or organs."

Watson says: "The cruel malady occurs most commonly in persons who exhibit, in other respects the signs of an unsound, or deranged, or debilitated system. It is more apt to fasten upon those who are pale, and asthenic, and upon individuals whose persons have been broken by advancing years."

Wood says: "Another state of system disposing to neuralgic attacks is debility. * * * The anemic condition is very favorable to it. Chlorotic females are extremely subject to neuralgic pains. * * * As to the state of the nerves, in those cases of neuralgia, if there be such, in which the true pathological condition is limited to the part where the pain is felt, we know absolutely nothing."

Now it will be observed that those authors all give positive evidence that *debility*, *anemia*, *chlorosis*, *and debilitating influences*, all give rise to a condition of system favorable to the development of neuralgia. But they stop just here, and take us no farther into the manner in which debility predisposes to the disorder in question. We join them in the opinion, that what we shall be pleased to term *idiopathic* neuralgia, is always based upon a depraved blood, and more or less debility. And in attempting to establish our theory we will, for the sake of convenience, make the arbitrary condition or classification of *idiopathic* and *symptomatic* neuralgia. But in truth all neuralgia is symptomatic of other pathological conditions. Will also consider the subject negatively, or *a fortiori*, and endeavor to arrive at just conclusions in relation to the pathological condition that obtains in neuralgia, and also to harmonize this pathology with the long-established, well attested, and successful treatment of the malady in question.

It is a fact that no one will dispute who has had any experience in the treatment of the disorder in question, or who has faithfully perused the medical record of the past, that almost all obstinate attacks of neuralgia, have required, and *do* require tonic treatment for their permanent removal; and the most efficacious tonics are the mineral, especially the ferruginous tonics; and these as a general thing require to be combined with a vegetable antiperiodic, and tonic remedy; more especially are these remarks applicable to cases occurring in malarial districts of country. By a close examination of authorities, and a review of the treatment invariably adopted in obstinate neuralgia, it will be seen that the above remarks are strictly true, and in keeping with the experience of the past.

We shall look first to the blood for the primary seat of trouble in neuralgia. In the normal or healthy state, we find upon analysis, that the salines enter into the composition of the blood. We find, however, that the saline matter constitutes but comparatively a small portion of the circulating medium, while the albumen, fibrine, and iron, or red corpuscles, constitute by far the greater portion of the blood. Now if from any cause whatever, those normal constituents of the blood become deranged, or abnormal in quality or quantity, or both, the consequence is disease of *some* character. Thus far it is plain that we are right. Well, if from some debilitating cause, or other, the blood becomes depraved, and it is found in this condition to contain an excess of saline matter, or deficit of red globules, would it not be reasonable, would it not be true, that, we would have a circulation well fitted to irritate a nervous system? We aver that it would be both reasonable and true that a circulation of blood containing an excess of saline matter, either independent, or in consequence of, a deficit of iron, is a circulation that will even entail disease upon the solids, and give rise to irritation of the nervous system, and consequently pains of a nervous or neuralgic character, as well as to many other pathological phenomena.

Now from the above remarks, our views of the pathology of neuralgia would seem reducible to the following conclusions:—A deranged condition of the blood, which derangement consists in an excess or preponderance of saline over coloring matter, or red corpuscles; that this condition of things is brought about by debilitating influences, or causes, which deteriorate the blood, rendering it defective in iron, thin, watery, and consequently excessive in saline matter—that this preponderance of saline over coloring matter, (iron) gives rise to irritation of nervous system, manifested in nervous pain, without any attending inflammation, which we denominate *idiopathic* neuralgia. It also will be observed that we base this pathology upon, and harmonize it with, the form of treatment that has invariably performed permanent cures, and the form of treatment that is well attested and authenticated by the most eminent medical writers of the past.

We will in the next place notice some of the *symptomatic* cases, also the modifications, and peculiarities of the disease under consideration. We not unfrequently see persons suffering with *aches* and *pains* denominated neuralgic, that have their origin in many other than the pathological condition above set forth as the true pathology of the malady in question. I do not ignore the fact that any pain arising from irritation of brain or nervous system and not attended by inflammation, is neuralgia, according to the construction put upon the term by Wood and others. But granting this, only serves to verify, rather than disprove our position; for we only claim that the affection is caused by irritation. And those cases of the disorder that will not bear, or do not demand tonic treatment, are generally produced by some accidental occurrence, as in nephralgia when caused by a calculus in the kidney or ureter, or when a predisposition is excited into action by a carious tooth, or by any mechanical injury which may be accidently inflicted upon a nervous trunk or plexus of nerves. Such cases we, for reasons afore mentioned, denominate *symptomatic.* They generally for their removal demand depletives and narcotics, especially the latter.

The disorder, as aforesaid, frequently assumes a remittent or intermittent character, especially is this applicable to those attacks which occur in malarial districts. But this fact only serves to still further substantiate our views as above recorded; for the malarial poison deteriorates the quality of the blood as surely and speedily as any other known agent. But it is not solely due to the malarial influence that the affection is paroxysmal; for in *that*, is only evinced the universal, and I may say immutable characteristic of irritation, which has *always* a disposition to intermissions, remissions, and exasperations. In those cases ferruginous tonics, in combination with vegetable antiperiodic medicines are required for the permanent removal of the difficulty.

We have done with the pathology of neuralgia, and if it be correct it will certainly direct in a more sure and successful path, those who have the painful and unbearable difficulty to deal with and treat. Whether it be the correct pathology or not, we can certify to one thing, which is this: that we invariably address our remedies, when called to a case of the disorder, to the pathological conditions above recorded; and we have quite recently permanently cured a number of cases which have harassed the patients from one to three years.

Will here report the particulars of one case, in order to give our plan of management when called to an obstinate case.

Was called in the month of January, to Mrs. H., the mother of two children, and aged about thirty years. She is of the nervo-bilious temperament. Said she had suffered more or less with an obstinate pain in the right side of the head for the past three years. Had been treated by Dr. W., Dr C., and Dr.—we can't say who else, and that none of them had even palliated the trouble for her. Her physical appearance evinced an anemic state of system; and upon closer examination she was found to be suffering with the most positive general debility, having in attendance all those functional derangements characteristic of that condition.

Diagnosis; Hernicrania: Therapia:

| | | |
|---|---|---|
| ℞ | Chiniodine, | 24 grs. |
| | Pulvis. Au. Cap. | 5 grs. |
| | Strychnia, | 1 gr. |

Mix—fiat. pil. No. 10 Dose—a pill before each meal.

After using the above sufficiently long to break down the paroxysms, and give the patient relief, we prescribed the following:

| | | |
|---|---|---|
| ℞ | Quevennes Iron, | 60 grs. |
| | Quinine, | 60 grs. |
| | Ext. Hyosciamus, | 40 grs. |
| | Pulvis. Aun. Cap. | 20 grs. |

Divide into 40 pills: dose—a pill after each meal, and to be continued until completely relieved of debility.

The patient did well from the commencement of the treatment, and is now restored to perfect health, at least as far as neuralgia is concerned.

The above is a fair specimen of our treatment when called to a case of, what we have termed in the present article, *idiopathic* neuralgia; and we can truthfully say, so far as our limited experience extends, we have *invariably* met with the *best* of success, having never failed to afford permanent relief in such cases.

